# Recent innovations in endodontic irrigation and effects on smear layer removal: an ex-vivo study

**DOI:** 10.1007/s00784-025-06387-1

**Published:** 2025-05-24

**Authors:** Ayfer Atav, Alessio Zanza, Aysenaz Gunes, Luca Testarelli, Massimo Galli, Qorri Erda, Michela Relucenti, Orlando Donfrancesco, Gianluca Gambarini

**Affiliations:** 1 Atlas University, Faculty of Dentistry, Department of Endodontics, Istanbul, 34408 Turkey; 2 Sapienza University of Rome, Department of Oral and Maxillo-Facial Sciences, Via Caserta 06, 00161 Rome, Italy; 3 İstinye University, Faculty of Medicine, Stem Cell and Tissue Engineering, Istanbul, 34396 Turkey; 4 Department of Dentistry, Faculty of Medical Sciences, Albanian University, Tirana, Albania; 5 Sapienza University of Rome, Department of Anatomy, Histology, Forensic Medicine and Orthopaedics, Section of Anatomy, Electron Microscopy Unit, Laboratory “Pietro M. Motta”, 00161 Rome, Italy; 6 Department of Life Science, Health and Health Professions, Link Campus University, 00165 Rome, Italy

**Keywords:** Endodontics, iVac, Irrigation, Laser, SEM

## Abstract

**Objectives:**

To evaluate the cleaning efficacy of different irrigation activation techniques in removing smear layers from root canals.

**Materials and methods:**

Ninety lower premolars with straight root canals were assigned to six experimental groups (*n* = 15 each): control group, conventional irrigation, passive ultrasonic activation (PUI), distilled water laser-activated irrigation (LAI), PulpSucker irrigation, and iVac irrigation. Each canal was shaped to size 30/04 and irrigated with 5% NaOCl. The final rinse was performed according to the experimental group. After chemo-mechanical procedures, the teeth were split longitudinally and subjected to scanning electron microscopic (SEM) analysis for each root canal third (coronal, middle, and apical). The presence of smear layer was evaluated using a 5-grade scoring system at 500× and 1000× magnification. Following the Shapiro-Wilk test, data were statistically analyzed using the nonparametric Kruskal-Wallis test, followed by the post-hoc Dunn test with Bonferroni correction (α = 5%), to compare the effectiveness of smear layer removal. The Friedman test and post-hoc Wilcoxon signed-rank test with Bonferroni correction (α = 5%) were performed to assess significant differences in smear layer removal among the different tooth thirds.

**Results:**

Activated irrigation techniques significantly outperformed conventional irrigation (*p* <.05), with the iVac technique demonstrating the best results in smear layer removal in the apical third. LAI and PUI showed comparable results across all tooth thirds. Significant differences in cleaning efficacy were observed among the different tooth thirds within each experimental group, with the apical third exhibiting the highest presence of smear layer.

**Conclusion:**

Within the limitations of the study, irrigant activation demonstrated higher efficiency in smear layer removal from root canal systems compared to conventional irrigation techniques. iVac showed the best cleaning performance in each third, particularly in the apical third.

**Clinical relevance:**

iVac technology offers significant potential for improving clinical outcomes.

## Introduction

The target point of the chemo-mechanical disinfection procedures in endodontics could be divided into organic and inorganic materials [1]. The first one is mainly represented by vital and/or necrotic tissues, bacteria, bacteria by-products, biofilm, inflammatory cells, and organic debris resulting from the breakdown of pulp tissue. On the other hand, the inorganic material is represented by dentin debris created during the instrumentation of dentinal walls. For the first time in 1975, McComb and Smith evidenced the smear layer concept in endodontics and defined it as a layer of debris created during the instrumentation of the root canal system [2]. This layer consists of both organic and inorganic materials, including dentin particles, remnants of the pulp tissue, and microbial elements, and it can impact the effectiveness of endodontic treatments by obstructing the penetration of irrigants and sealers​ into dentinal tubules. It is divided into two parts: the surface smear layer, which is a thin, superficial layer of debris that coats the dentinal walls of the root canal and consists of fine particles of organic and inorganic material, including dentin, pulp tissue remnants, and microorganisms, and the smear plugs, which are more densely compacted particles that become lodged within the dentinal tubules, effectively occluding them. In the light of above, the irrigation procedure is crucial to guarantee the optimal removal of organic and inorganic materials from endodontic systems [2].

Over the years, several endodontic irrigants have been proposed, however, to date the gold standard is represented by sodium hypochlorite (NaOCl) and ethylenediaminetetraacetic acid (EDTA) [1]. NaOCl is the most widely used irrigant due to its potent antimicrobial properties and ability to dissolve organic tissue. It is typically used in concentrations ranging from 0.5 to 6%. NaOCl effectively targets and eliminates bacteria, biofilm, and organic debris within the root canal system. Its tissue-dissolving capabilities help in removing pulp remnants and other organic materials, enhancing the overall cleaning efficiency of the root canal [3, 4]. EDTA is a chelating agent primarily used to remove the inorganic component of the smear layer and to open dentinal tubules. It is usually employed in a 17% concentration. EDTA works by binding to calcium ions in the dentin, facilitating the removal of mineralized debris and smear layer. This process increases the permeability of the dentin, allowing for better penetration of disinfecting agents and sealers [3, 4]. Therefore, the combination of NaOCl and EDTA is often used sequentially in root canal therapy. NaOCl is used first to dissolve organic tissues and disinfect the canal, followed by EDTA to remove the smear layer and ensure the canal walls are free of debris and open for final irrigation and obturation. This synergistic approach maximizes the effectiveness of the cleaning and disinfection process, ensuring a more thorough and successful endodontic treatment.

Various irrigation activation techniques have been developed to enhance the effectiveness of root canal cleaning. These techniques aim to improve the distribution and agitation of irrigants within the canal system, thereby increasing the removal of debris and smear layers. Passive ultrasonic irrigation (PUI) is one of the most widespread activation methods [5]. The method employs an ultrasonic device with a thin, flexible tip that vibrates at high frequencies to enhance the penetration and agitation of irrigants within the root canal. These vibrations create cavitation and acoustic streaming effects, which aid in removing debris and bacteria from canal walls and irregularities [6, 7]. Another common activation method is the laser-activated irrigation (LAI). It is a relatively recent technique that utilizes laser energy to improve the cleaning efficiency within the root canal system. This technique relies on the rapid heating of the irrigant by Er: YAG or Er, Cr: YSGG lasers, which produces optic cavitation [8, 9]. The energy produced induces a photomechanical effect, leading to the generation of shock waves that facilitate the removal of debris and the disruption of biofilms [10].

Recently, a new Er, Cr: YSGG laser system, EdgePRO™ (EdgeEndo, Albuquerque, New Mexico, USA), has been introduced. According to the manufacturer, it utilizes advanced laser, light, and sound technologies to safely and effectively perform specific endodontic applications. The EdgePRO laser operates in three different procedures according to users’ preferences: the “water endo” program, in which the final rinse is performed with the activation of distilled water; the “hybrid technique”, in which the final rinse is performed with the activation of distilled water, EDTA and NaOCl; the “Mid-Root” program, in which the final rinse is performed with the activation of NaOCl, EDTA, and NaOCl again. To date, there are no data available on the activation with distilled water without other solutions.

In addition to the activation mentioned above techniques, in the last years, two different devices have been introduced to enhance the chemical disinfection of the root canal system: the PulpSucker (PlanB Dental, Goleta, CA, USA) and the iVac (Pac-Dent, Brea, CA, USA). The Pulp Sucker is a device designed to facilitate the removal of pulp tissue and debris from the root canal. It utilizes an automated, continuous, multi-cannular irrigation of root canal system by creating negative irrigation pressures in the pulp chamber, thereby passively drawing copious volumes of fresh irrigating solutions out of the ends of the cannulae in a completely controlled manner. For each type of tooth it has a kit containing the whole equipment set for final irrigation such as; SmearOff syringe (17% EDTA + CHX), staging assembly, staging placement device, VacuSeal, Clor-XTRA Plus(enhanced 8.0% NaOCl). The IVac is another relatively recent innovation that incorporates a tip manufactured of high-performance medical-grade polymer with a piezoelectric ultrasonic handpiece to improve the removal of debris and irrigant exchange within the root canal thanks to the irrigant agitation and aspiration with apical negative pressure.

Previous studies have shown varying degrees of success with conventional irrigation, LAI, and PUI. Still, only 1 article was published on the effectiveness of irrigant activation with iVac and no data are available on PulpSucker and EdgePRO laser [11]. Therefore, a comprehensive comparison of their effectiveness in removing debris and smear layers is limited, thus, this study aims to fill this gap by systematically evaluating and comparing these activation techniques in extracted teeth through a scanning electron microscope (SEM) observation. The null hypothesis of this study was that there is no significant difference among the different irrigation activation and agitation systems, including the control group, in their effectiveness at removing the smear layer.

## Materials and methods

The manuscript of this laboratory study has been written according to Preferred Reporting Items for Laboratory Studies in Endodontology (PRILE) 2021 guidelines [12].

This study was approved by the ethical committee of the Faculty of Medical Sciences, Albanian University (Ethical committee approval Nr.171 Prot Dt.13.05.2024) and was conducted in accordance with the ethical principles outlined in the Declaration of Helsinki. Written informed consent was acquired from all donors and their confidentiality and anonymity were strictly maintained throughout the study.

### Experimental groups allocation

The sample size calculation was performed based on previous data reported in published studies comparable in terms of aims and methodology, using G*Power v3.1 (Heinrich Heine, University of Düsseldorf, Düsseldorf, Germany) by selecting the ANOVA test and setting an alpha-type error of 0.05, a beta power of 0.90, and an effect size of 0.80 [13, 14]. A total of 10 samples per group were indicated as the ideal size required for noting significant differences. However additional 5 samples per group were added to compensate for unexpected results with PulpSucker and EdgePRO laser since there was no data in the literature regarding their ability to remove the smear layer, and only 1 article was found in the literature reporting on iVac.^11^ According to this, a total of 90 freshly extracted mature human teeth were selected, 15 for each of the 6 groups and randomly allocated as follows:

- Group 1: control group.
- Group 2: conventional irrigation group.
- Group 3: LAI group.
- Group 4: PUI group.
- Group 5: PulpSucker group.
- Group 6: iVac group.

### Tooth selection

The 90 freshly extracted human teeth were selected according to inclusion and exclusion criteria. Teeth must abide by the following characteristics: premolar extracted due to orthodontics reasons, premolar with a single canal with a curvature < 15° according to Schneider’s method [15], maximum transversal diameter ranging from 0.2 mm to 0.3 mm for the apical third, 0.3 mm and 0.5 mm for the middle third and 0.8 to 1.0 mm for the coronal third, total tooth length ranging from 20 mm to 24 mm, mature apex, no previous endodontic or intracanal treatments, no calcification or intracanal pulp stone, no internal or external root resorption, giver > 18 years old and < 35 years old, no root fracture or microcracks, extraction performed no more than 6 months earlier and storage in a solution of physiologic saline at 4 °C. The anatomical characteristics were evaluated by a Cone-Beam Computer Tomography (CBCT) after the extraction with the onDemand 3D Dental software (Cybermed, Seoul, Korea) programme.

### Tooth Preparation and shaping procedure

The root canal of each tooth was apically sealed with melted wax to close the major apical portal of exit to prevent the extrusion of irrigants and to simulate in vivo conditions [16]. Access cavity was performed using a rounded diamond bur performing a traditional access cavity, according to the classification proposed by E. J. N. L. Silva et al. [17]. After the patency check with a k file #10, the teeth were shaped with Xp-endo (FKG Dentaire SA, La Chaux-de-Fonds, Switzerland) protocol first creating the glide path with 15/0.04 file and then shaping was continued with XP-endo Rise (FKG Dentaire SA, La Chaux-de-Fonds, Switzerland) at 1000 rpm and 1 Ncm torque. The whole shaping procedures were performed in a sodium hypochlorite (NaOCl) bath with a concentration of 5%. 5 ml of NaOCl was used between each instrument. After shaping procedures, teeth were numbered and randomly divided into 6 groups according to final irrigation protocol with computer assisted randomization programme.

### Final irrigation protocols

The final irrigation protocol was performed differently according to each group as follows:

- **Group 1 (control group)**: No final rinse and no additional agitation of the irrigant were performed after shaping procedures.
- **Group 2 (conventional irrigation)**: The root canals were flushed with 5 mL 17% EDTA for 1 min, followed by 1 min irrigation with 5 mL 5.25% NaOCl by using a 30-gauge irrigation needle (Ultradent, South Jordan, UT, USA) placed 1 mm short of the working length (WL). The NaOCl irrigation was performed after a distilled water rinse.
- **Group 3 (LAI)**: after shaping procedures, the canal was rinsed with distilled water and the chamber was flooded. The “Water Endo” program of the EdgePRO laser was selected and tip #1 (yellow coded, 200 μm and 25 mm length) was placed 1 mm short of the WL. With a slow circular motion, the tip was retracted 1 mm per second while activating the laser up to the canal orifice. The laser was not active during the downward strokes. This procedure was repeated four times in each canal. The canals were then dried with paper points and tip #1 was placed 1 mm short to the WL for the “Dry and Disinfection” procedure by selecting the “Dry and Disinfection” preset. With a slow circular motion, the tip was retracted 1 mm every second while activating the laser up to the canal orifice. This procedure was repeated four times in each canal. EdgePRO laser optical characteristics are illustrated in Table 1.
- **Group 4 (PUI)**: an Ultra X Silver insert #20/02 tip was inserted 1 mm from the apex and 5 mL 17% EDTA activated for 1 min at 45 kHz using a portable ultrasonic device (Ultra X, Eighteeth, Changzhou, China) for 1 min. After aspiration and distilled water rinse, 5 mL 5.25% NaOCl, was activated with the ultrasonic device for 1 min.
- **Group 5 (PulpSucker)**: Teeth were rinsed with water spray, dried with the air/water syringe, and isolated. Roots and apex were covered with melted dental wax. Crowns were coated with VacuSeal in a ring-like fashion and light-cured before placement of the stage part of the kit. With the help of the placement tool Pulp Sucker stage was inserted into the prepared VacuSeal. VacuSeal was injected all around the tooth and light-cured, and flexible stage skirts eased the process both for injection and for light-curing steps of the VacuSeal. Cannulas were measured and stops were adjusted 1 mm short of working length. Placed into the root canals. VacuSeal was applied and light-cured to hold cannulas and Top Plate stable in their places on the stage. The Vacuum Line was plugged in to enable the evacuation system. By using the empty syringe, the air was transfused into the Chlor-Xtra Plus bag and the flow of the solution was checked. Then, the Chlor-Xtra Plus bag was injected into the system. Fluid from the NaOCl bag ran through the system until the bag was empty. After turning off the lever, suction took the remnant of solution from the canals while the Vacuum Line part was still attached to the suction. Pulp sucker closed system staging devices were adapted to access cavities of teeth according to the manufacturer recommendations as mentioned above. The whole recommended solution in the premolar kit was used for final irrigation. A new premolar pulp sucker kit was used for each tooth.
- **Group 6 (iVac)**: Ultrasonic agitation with iVac system: 5 mL 17% EDTA and 5.25% NaOCl were activated using the iVac system (0.35 tip), coupled in Woodpecker Piezo Cavitron Ultrasonic Scaler (Guilin Woodpecker Medical Instrument Co., Ltd, Guilin, China). The iVac tip was inserted 1 mm of the working length. Five milliliters of each solution (sodium hypochlorite, EDTA) were used, and agitation was performed in three cycles of 20 s, totaling 1 min of agitation.

**Table 1:** Optical characteristics of edgepro laser

Optical characteristics	
Laser classification	IV (4)
Medium	Er, Cr: YSGG
Wavelength	2.78 μm
Mode	Free-running Pulsed
Frequency (Pulse Rate)	5–50 Hz
Average Power	0.1–2.0 W
Pulse Energy	10–100 mJ
Pulse Duration	60 µs
Fiber Tip Diameter Range (Spot size)	200–500 μm
Aiming Beam	625–670 nm (red) laser, 1mW max (Laser Class 1)

#### Sample Preparation for SEM observations

After the final irrigation procedures, the canal was dried with sterile paper points and a custom gutta-percha point was inserted in it to facilitate the sectioning procedures. A continuous longitudinal groove was done in a buccolingual direction passing through the center of the apical foramen and to the center of the occlusal perimeter of the access cavity design. The longitudinal groove was performed with a Frios diamond-cutting disk (Microsaw; Dentsply Friadent, Mannheim, Germany) mounted on a surgical dental handpiece cooled with water until the orange color of the gutta-percha was slightly visible in transparency. The actual and definitive split was done with a chisel to avoid intracanal contamination.

#### SEM observation and scoring

**Fig. 1:**
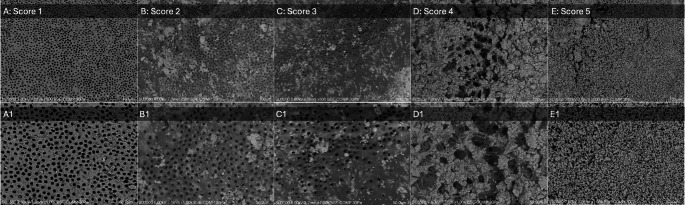
Graphical illustration of the scale used to evaluate the debris and smear layer removal. (**A**) Score 1, magnification x500; (**B**) Score 2, magnification x500; (**C**) Score 3, magnification x500; (**D**) Score 4, magnification x500; (**E**) Score 5, magnification x500. (A1, B1, C1, D1, and E1) Sections of the images A, B, C, D, and E at a magnification of ×1,000

Samples were observed without any coating procedure. Specimens were carefully dried at room temperature by resting on a filter paper for 1–2 min. Then, they were mounted onto aluminum stubs by carbon tape and placed in an oven at 37 ◦C for 2 h before observation with the variable pressure SEM Hitachi SU–3500 (Hitachi, Tokyo, Japan). The observations were performed at operating conditions of 30 Pa and 5.0 to 7.0 kV. Images were captured at several magnifications between x30 and x1000. Each root half was first observed at low magnification (×30) to obtain a general overview of the sample and then divided into three thirds (coronal, middle, and apical) in order to evaluate the presence of smear layer in each portion. In each third, image acquisition on the most significant zones was performed at a magnification range from x250 to x1000 to assess the presence of the smear layer. Two blind operators evaluated the images, scoring them according to the scoring system proposed by Caron et al. [14] (Table 2; Fig. 1). In order to assess the intraobserver reproducibility and consistency, Cohen’s kappa was calculated, resulting in a value of 1, stating the perfect agreement between the two observers.

**Table 2:** Description of the scoring system defined by caron et al. [14]

Scoring system	Description
Score 1	no smear layer and dentinal tubules almost completely open
Score 2	small amounts of scattered smear layers and dentinal tubules almost completely open
Score 3	thin smear layer and dentinal tubules partially open (characteristic image of the crescent)
Score 4	partial covering with a thick smear layer
Score 5	total covering with a thick smear layer.

Statistical analysis was performed using Python (Version 3.12.5, Python Software Foundation, Beaverton, OR, USA) within the Visual Studio Code integrated development environment (Version 1.92.2, Microsoft Corporation, Redmond, WA, USA). The non-nonparametric Kruskal-Wallis test was used to compare the effectiveness of smear layer removal among the experimental groups (for each tooth third) and the post-hoc Dunn test with Bonferroni’s correction was used for pairwise comparisons, with a significance level set at α = 0.05. To evaluate whether there were significant differences in the effectiveness of smear layer removal among different tooth thirds (apical, middle, coronal) within each experimental group, the Friedman test and the post-hoc Wilcoxon signed-rank test with Bonferroni’s correction were performed, with a significance level set at α = 0.05.

## Results

**Fig. 2:**
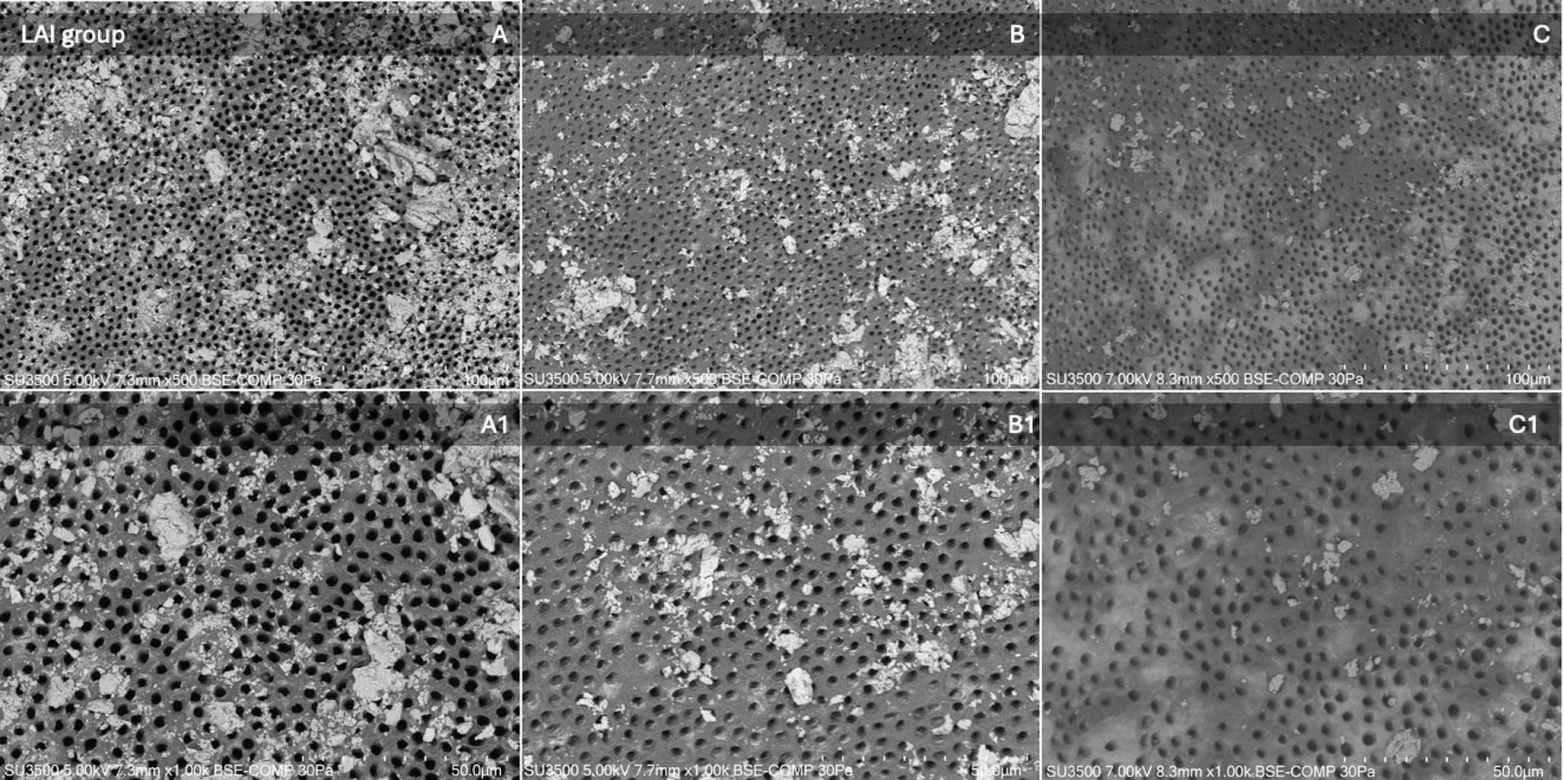
SEM observations of the LAI group. Images on the top line represent respectively (**A**) apical third, (**B**) middle third, and (**C**) coronal third at a magnification of x500. Images on the bottom line represent the above-mentioned thirds at a higher magnification of x1000. The SEM parameters (kV, pressure (Pa), imaging mode (backscattered electron composition, BSE-COMP), and µm scale) are shown at the bottom of each image

**Fig. 3:**
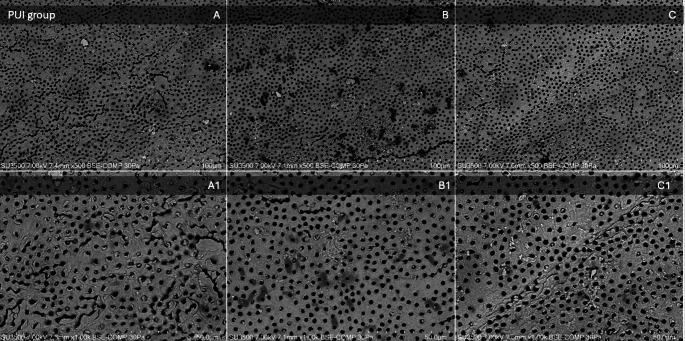
SEM observations of the PUI group. Images on the top line represent respectively (**A**) apical third, (**B**) middle third, and (**C**) coronal third at a magnification of x500. Images on the bottom line represent the above-mentioned thirds at a higher magnification of x1000. The SEM parameters (kV, pressure (Pa), imaging mode (backscattered electron composition, BSE-COMP), and µm scale) are shown at the bottom of each image

**Fig. 4:**
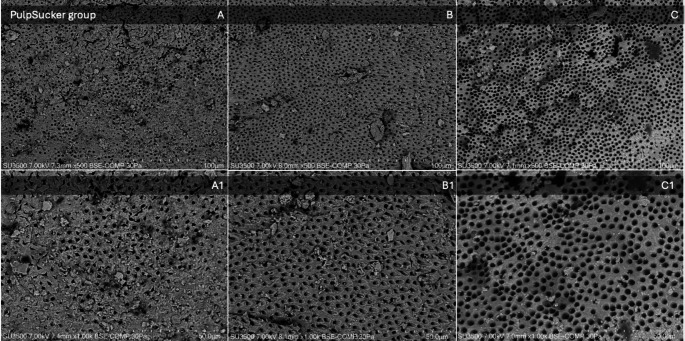
SEM observations of the PulpSucker group. Images on the top line represent respectively (**A**) apical third, (**B**) middle third, and (**C**) coronal third at a magnification of x500. Images on the bottom line represent the above-mentioned thirds at a higher magnification of x1000. The SEM parameters (kV, pressure (Pa), imaging mode (backscattered electron composition, BSE-COMP), and µm scale) are shown at the bottom of each image

**Fig. 5:**
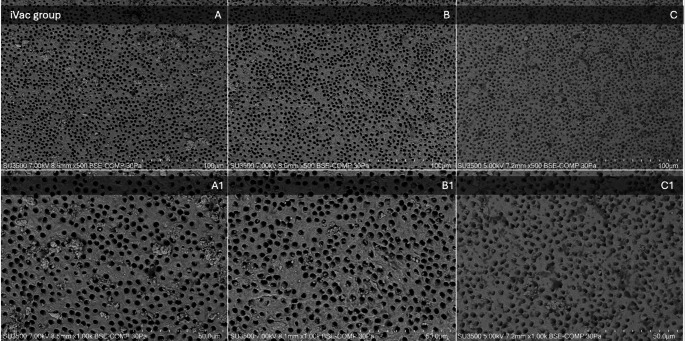
SEM observations of the iVac group. Images on the top line represent respectively (**A**) apical third, (**B**) middle third, and (**C**) coronal third at a magnification of x500. Images on the bottom line represent the above-mentioned thirds at a higher magnification of x1000. The SEM parameters (kV, pressure (Pa), imaging mode (backscattered electron composition, BSE-COMP), and µm scale) are shown at the bottom of each image

Mean scores, median, and standard deviations (sd) for smear layer removal in the apical, middle, and coronal thirds of each group are listed in Table 3. Images of SEM observations of groups are shown in Figs. 2, 3, 4 and 5.

**Table 3:** Mean, median, and standard deviation (sd) comparison for smear layer removal in the apical, middle, and coronal thirds of each group. Same superscript letters indicate a statistically significant difference between groups within the same root Canal third (*p* <.05) (columns). Same subscript numbers indicate a statistically significant difference between Canal thirds within the same group (*p* <.05) (rows)

	Apical third			Middle third			Coronal third		
**Group**	*mean*	*median*	*sd*	*mean*	*median*	*sd*	*mean*	*median*	*sd*
*Control group*	5.00^a, f,g, h^	5	0.00	4.40^a, c,d, e,f^	4	0.51	4.40^a, b,c, d,e, f^	4	0.51
*Conventional irrigation*	4.87^b, c,d, e^_1_	5	0.34	4.20^b, g,h, i,l^_1_	4	0.56	3.07^b, g,h, i,l^_1_	3	0.59
*LAI*	2.33^a, b,i^_1_	2	0.62	2.13^c, g^_2_	2	0.64	1.20^c, g^_1,2_	1	0.41
*PUI*	2.23^f, c,l^_1,2_	2	0.63	1.20^d, h^_1_	1	0.41	1.13^d, h^_2_	1	0.35
*PulpSucker*	3.27^g, d,m^_1_	3	0.59	2.33^e, i,m^_1_	2	0.62	1.27^e, i^_1_	1	0.46
*iVac*	1.13^h, e,i, l,m^_1_	1	0.32	1.13^f, l,m^_2_	1	0.35	1.07^f, l^_3_	1	0.26

Regarding the statistical comparison of the effectiveness of smear layer removal with experimental irrigation techniques across different tooth thirds, iVac showed the best performance in each third, particularly in the apical third, where it demonstrated a statistically significant difference (*p* <.05) compared to other irrigation techniques. In the middle third, iVac, LAI, and PUI showed the best performance without any statistically significant difference among them (*p* >.05). Differently, in the coronal third, there were no statistically significant differences among iVac, LAI, PUI, and PulpSucker (*p* >.05). The conventional irrigation without any agitation/activation demonstrated the worst performances in all tooth thirds with a statistically significant difference (*p* <.05), except for the comparison with PulpSucker in the apical third, where the two irrigation systems did not show any statistically significant difference (*p* >.05). The entire statistical analysis of the comparison between the different irrigation techniques is shown in Table 3.

Regarding the statistical comparison of the effectiveness of smear layer removal in different tooth thirds within the same experimental group, the apical third was the worst cleansed zone with a statistically significant difference (*p* <.05) in comparison to the middle and coronal thirds within each experimental group except for LAI, which guaranteed the same smear layer removal in the apical and middle thirds, and iVac, which showed the same effectiveness of smear layer removal in each third (*p* >.05). Regarding conventional irrigation and PulpSucker, the effectiveness of smear layer removal was statistically significant for each third, with decreasing effectiveness from the apex to the coronal third (*p* <.05). The entire statistical analysis of the comparison of the effectiveness of smear layer removal in different tooth thirds within the same experimental group is shown in Table 3.

## Discussion

As extensively demonstrated, debridement and chemical disinfection of root canal systems provided by shaping and cleaning procedures are crucial factors in determining the long-term success of endodontic treatments [1, 18]. The prevailing paradigm posits that irrigants are responsible for the majority of the cleaning and disinfection within the root canal system, while instrumentation primarily serves to provide access to the apical anatomy [1]. This concept arises from the fact that endodontic instruments leave numerous untouched areas within the complex endodontic system due to the presence of anatomical intricacies [19, 20]. For this reason, in the last decades several activation and agitation devices have been introduced to increase the chemical power of irrigants, both in terms of antimicrobial activity and inorganic tissue removal [1]. Despite the plethora of research published on PUI and laser activation, there are few articles on a comprehensive comparison of different irrigation systems, resulting in fragmented knowledge which may result in an oversimplification of the investigated phenomena. Therefore, the current study aimed to comprehensively compare the effectiveness of removing smear layers of several devices, including new technologies (iVac, EdgePRO laser and PulpSucker), of which the data are scarce.

Regarding the primary objective of this study—comparing the cleaning effectiveness of activated/agitated irrigation techniques with conventional irrigation methods—the results and statistical analysis demonstrated that all agitation or activation systems performed significantly better than traditional irrigation in terms of smear layer removal across all thirds of the root canal (apical, middle, and coronal). Conventional irrigation only showed a significant difference compared to the control group (no final rinse) in the coronal third, highlighting the crucial role of irrigant activation during chemo-mechanical disinfection of root canals. The reduced efficacy of irrigants delivered via syringe without further activation may be attributed to the inability of conventional irrigation to effectively disrupt biofilms or planktonic bacteria in non-instrumented areas, due to the low delivery pressure. Such reduction is also related to the limited mechanical preparation in the apical and middle thirds compared to the coronal third. This finding is consistent with several previous studies [21–23].

Regarding the comparison of smear layer removal among the experimental groups, iVac demonstrated the highest cleaning effectiveness, with a median score of 1 in each root canal third. This result revealed a statistically significant difference in iVac activation compared to PUI, LAI, and PulpSucker in the apical third, and compared to PulpSucker in the middle third, whilst no statistically significant differences have been found in the coronal third. There were no statistical differences among the four activation or agitation methods in the coronal third. The increased cleaning efficiency of iVac in comparison to other irrigation techniques is probably due to its combination of different techniques which contributes to the increased fluid dynamics and consequently chemical disinfection. The iVac is a system that merges three different agitation techniques: the piezoelectric ultrasonic activation, which—thanks to the oscillation frequencies within the range of 30 kHz (according to the manufacturer)— can provide transient cavitation and microstreaming effects; the negative pressure, by which the irrigants flow from the pulp chamber to the apical limit without extruding beyond the foramen; the concomitant irrigation, by which an abundant, continuous renew of the volume of irrigants is supplied, ensuring their sustained efficacy. The results of the current study are partially consistent with the recent data published by de Oliveira Neto et al. [11]. In their article, the authors investigated the cleaning efficacy and the organic tissue dissolution capacity of iVac and PUI in lower premolars with straight root canals. They found no statistically significant differences between the above-mentioned experimental groups among each root canal third, even in the apical third, observing overlapping data [11]. The differing results with the current study can be attributed to variations in methodology, particularly regarding the final shape and dimensions of mechanical preparation. De Oliveira Neto et al. prepared the root canals up to size #50 with a 0.05 mm taper, while in the present study, the canals were shaped up to size #30 with a taper of 0.04 mm. This increase in preparation volume could facilitate the easier insertion of the non-flexible stainless-steel tip used for PUI, allowing it to oscillate in a larger space easily. However, considering the principles of minimally invasive shaping, a preparation size of #50.05 may not be clinically reliable especially in complex anatomies, as it could lead to iatrogenic errors and excessive weakening of the structural integrity of endodontically treated teeth [24]. Considering this, the iVac system could be considered more effective in smear layer removal in most cases than PUI in cleaning efficacy in the apical third.

Despite the different median values, which seemed to demonstrate the greater performance of LAI and PUI compared to PulpSucker in removing smear layer, the statistical analysis found no statistically significant difference among the methods mentioned above. This conclusion is not consistent with the results published by Liang et al. [25]. In this systematic review, the authors affirmed that the irrigation efficacy significantly increased in LAI compared with that in PUI. Nevertheless, the systematic review took into consideration two specific LAIs (PIPS and SWEEPS), which are different lasers from the one used in this study. Moreover, other articles demonstrated no statistically significant differences between conventional LAI, PIPS, and PUI in terms of the percentage of smear removed, which is consistent with the results of the current study [26, 27]. Thus, a definitive conclusion regarding the actual comparison between LAI and PUI cannot be drawn due to the high heterogeneity of methodologies, types of laser used, and their settings, and further research is needed to assess this point and to clarify whether the use of one irrigant activation technique is clinically superior to another in terms of long-term endodontic success. In this study, the laser used was an Er, Cr: YSGG system, and the final activated rinse was performed using distilled water, without any other solutions (e.g., NaOCl or EDTA), following the “Water Endo” preset available on the laser. The results appear promising, as the LAI activation, without the addition of NaOCl or EDTA, showed comparable cleaning efficacy to the other techniques. Replacing solutions like NaOCl and EDTA in the final stages of activation could lead to a significant reduction in adverse events related to solution extrusion into periapical tissues [28]. Additionally, given the complete safety of water compared to other solutions, it is possible to position the activating source closer to the apical third, allowing for improved cleaning in that region. Significant benefits might also be observed regarding the adhesion strength of posts and post-endodontic restorative materials, as it has been demonstrated in the literature that solutions like EDTA and NaOCl compromise adhesion [29, 30]. From a qualitative analysis of the SEM images, LAI activation with water successfully opened dentinal tubules in all thirds when exposed. In the visible tubules, no scattered material was observed inside, although it slightly underperformed in removing the entire surface smear layer. In contrast, other activation methods, such as PUI and iVac, completely removed the surface smear layer but sometimes failed to remove the smear plugs inside the dentinal tubules. Likely, the use of EDTA (which was not used in the LAI group) played a crucial role in the removal of the surface smear layer. Further research is needed to optimize debris and smear layer removal with LAI based on distilled water, possibly by modifying the protocol to include final syringe rinsing with varying amounts of EDTA or NaOCl.

Regarding the comparison of LAI and PUI with PulpSucker, the data are consistent with studies suggesting that negative pressure irrigation systems are comparable to ultrasonic and laser-activated irrigation techniques [31–33]. The hypothesis is that, despite the absence of cavitation effects, negative pressure irrigation ensures a constant and continuous flow of fresh irrigants, providing a larger volume while reducing the risk of extrusion. Based on the results presented, the combination of ultrasonic activation and negative pressure irrigation, as demonstrated by the iVac system, achieved the highest cleaning efficacy. LAI and PUI are also simpler to clinically use because they do not need to ensure a vacuum in the pulp chamber, especially in teeth with multiple canals. In the current study, this disadvantage was reduced by selecting teeth with a single canal.

The statistical analysis comparing the effectiveness of smear layer removal in different tooth thirds within the same experimental group confirmed that the apical third is the area where debridement is more difficult due to the smaller reservoir volume and its intrinsic distance from the coronal portion which hinders irrigant access [34–36]. This is the reason why activation is currently the recommended procedure, which is mainly aiming at improving cleaning and disinfection of the apical third. Ideally, activation should provide excellent results even in the apical area. In our study, this conclusion applies only to LAI and iVac, which showed no statistically significant differences between the apical and middle thirds. This could be attributed to the ability of these techniques to deliver irrigants more effectively to the apical region than other methods, ensuring greater cleaning efficacy. Those results are consistent with data published in the literature [21].

This research is based on an ex-vivo observation through scanning electron microscopy to determine the effectiveness of different irrigation techniques to remove smear layers. Over the years, despite the plethora of articles published following this method, several concerns have been raised about its methodological limitations [37]. The most common points have been: the introduction of artifacts during the dehydration of the specimens and coating process which may interfere with observations, the destructive methodology of SEM observation, the unknown prior status of the root canal, the not genuinely random selection of the observed spots and the lack of blinding bias, the possibility to confound sclerotic dentine with no open dentinal tubules, the absence of calibration within the observers [37, 38]. In light of the above, the current study was designed to limit those concerns. The SEM used for the observation was a variable pressure SEM Hitachi SU–3500, which does not require any coating procedure or hard dehydration before observation, thus, the risk of artifacts due to the sample preparation procedures was eliminated. Regarding the problem of confounding sclerotic dentine with no open dentinal tubules due to a reduced cleaning efficacy of irrigants, it was overcome by selectin teeth from a specific population filtered by age (18–35 years old), reducing as much as possible the probability of sclerosis, since it has been demonstrated that it is a phenomenon linked to the elder age [39]. Moreover, the risk of the not genuinely random selection of the observed spots and the lack of blinding bias was overcome by measuring the intraobserver reproducibility and consistency with Cohen’s kappa, and the SEM operator, which selected the images for the scoring, was blind to avoid any tendency of selecting relatively clean areas and the indication was to capture the most representative area of each specimen. Moreover, the experimental groups were not revealed to the SEM observer.

Nevertheless, SEM studies have inherent limitations, with the destructive nature of the method being one of them. In this regard, micro-CT examinations offer a better alternative. However, Micro-CT is unable to detect the presence of smear plugs or radiolucent materials inside dentinal tubules, and like SEM analysis, it is not free from methodological errors, particularly during critical phases such as data processing, segmentation, image overlay, and noise reduction [40].

Although the current study was conducted ex vivo and the observation of the smear layer took place in a controlled laboratory setting, direct clinical conclusions should not be drawn from these findings. As with other surrogate endpoints used in root canal irrigation studies, there is currently no evidence to suggest that the removal of dentine the smear layer directly improves the chances of healing apical periodontitis. Ex-vivo and in-vitro research is essential for laying the groundwork for future in-vivo clinical studies. Based on current knowledge, it is reasonable to assume that a higher number of patent dentinal tubules and a reduced amount of debris within the root canals create an ideal environment for proper canal obturation and successful endodontic outcomes. However, further clinical studies are strongly recommended to evaluate the actual relationship between debris and smear layer removal and the success of root canal treatments.

## Conclusion

Considering the limitations of the current study, the activation of irrigants proved to be an efficient method for removing smear layers from root canal systems and should be preferred over conventional irrigation systems during disinfection procedures. Among the techniques evaluated, the combination of ultrasonic agitation and negative pressure irrigation represented by the iVac group showed superior results. Further clinical studies are strongly recommended to understand the precise relationship between the recent innovations in endodontic irrigation and overall success of root canal treatments.

## Data Availability

No datasets were generated or analysed during the current study.
